# Diagnostic and prognostic value of serum Cys-C, retinol-binding protein, and ischemia-modified albumin in patients with coronary heart disease: A diagnostic accuracy study

**DOI:** 10.1097/MD.0000000000039415

**Published:** 2024-08-30

**Authors:** Youhua Yuan, Wenqian Tian, Xiaoxia Wei, Ya Zhu, Fengzhen Liu, Xiaohuan Zhang

**Affiliations:** a Department of Laboratory, Henan Provincial People’s Hospital, People’s Hospital of Zhengzhou University, People’s Hospital of Henan University, Zhengzhou, China.

**Keywords:** coronary heart disease, cystatin-C, ischemia, modified albumin, retinol-binding protein

## Abstract

The use of 3 biomarkers – cystatin-C (Cys-C), retinol-binding protein (RBP), and ischemia-modified albumin (IMA) – for the clinical classification and outcome of coronary heart disease (CHD) has not been adequately evaluated. We explored the serum levels of these 3 markers and evaluated their diagnostic and prognostic values in patients with CHD. This retrospective case–control study, conducted between June 2017 and June 2018, included 201 patients with CHD hospitalized at the Henan Provincial People’s Hospital and 127 healthy individuals from Henan Provincial People’s Hospital as controls. Cys-C, RBP, IMA levels, and other laboratory parameters in the 2 groups were determined, and patient outcomes were analyzed. Cys-C, RBP, and IMA levels were higher in the case group than in the control group (*P* < .05). Logistic regression analysis confirmed that these 3 biomarkers were independent risk factors for CHD. Each indicator has clinical significance in the diagnosis and prognosis of CHD, with RBP being the most significant. The AUC value for CHD detection using a combination of the 3 indicators was 0.783, and the sensitivity and specificity values were 78% and 74.6%, respectively. Simultaneous detection of Cys-C, RBP, and IMA could be an optimal method for early diagnosis and prognosis of CHD.

## 1. Introduction

Coronary heart disease (CHD) is caused by coronary artery stenosis or occlusion due to myocardial ischemic hypoxia, which triggers cardiac muscle cell necrosis and the development of heart disease. According to recent investigations, the incidence and mortality of CHD among urban residents in China are increasing annually, as is the use of interventional surgery for coronary events.^[1]^ The occurrence and outcomes of CHD are associated with various factors.^[2]^ Coronary atherosclerosis, myocardial ischemia, and necrosis are common underlying pathophysiological causes of heart disease. Atherosclerosis is currently considered a chronic inflammatory reaction that causes endothelial cell abnormalities, leading to structural and functional dysregulation of the endothelial vascular matrix. This, in turn, promotes the progression and rupture of atherosclerotic plaques and formation of thrombosis, causing arterial lesions in CHD.^[3,4]^

Coronary angiography (CAG) is the standard method for diagnosing CHD; however, it has drawbacks such as invasiveness, high cost, inconvenience, and the requirement for frequent follow-up visits. Consequently, CAG may be unsuitable for population-wide CHD screening.^[5]^ Therefore, investigators are still exploring reliable and simple biomarkers for the early detection of CHD in healthy people, which can help physicians identify patients at risk of a poor or even fatal outcome, design precision therapy for specific CHD subphenotypes, and monitor response to treatment.^[6–8]^

Cystatin-C (Cys-C) is a small-molecule cysteine protease inhibitor that is freely filtered by the glomerulus and metabolized after tubular reabsorption, thus a good indicator of renal function.^[9]^ In the last few decades, Cys-C has been shown to be associated with the regulation of inflammatory responses in vivo and has been closely linked to the occurrence and progression of atherosclerosis.^[10]^ Retinol-binding protein (RBP) is a single-stranded binding protein primarily synthesized in the liver that transports vitamin A (retinol) from the liver to other tissues. After a series of biological transformations, RBP increases retinol stability and prevents its oxidation, thereby maintaining a stable retinol concentration. Additionally, RBP improves retinol function^[11]^ and plays a crucial part in carbohydrate and lipid metabolism, metabolic syndrome, insulin resistance, vascular endothelial injury, atherosclerosis, and CHD.^[12]^ Myocardial ischemia-modified albumin (IMA) is a biochemical index that reflects ischemia and hypoxia of cells in the body. IMA is formed by terminal acetylation or deletion of the binding sites for metal ions at the amino terminus of serum albumin when cells are under stress, such as during ischemia and hypoxia.^[13]^ IMA can reflect ischemia within minutes of the development of necrosis. The American Food and Drug Administration has accepted IMA as a diagnostic biomarker for CHD.^[14]^

Therefore, Cys-C, RBP, and IMA are closely associated with the development and progression of CHD. Although numerous studies have reported a relationship between CHD and these 3 biomarkers, they have been evaluated individually.^[15]^ However, to our knowledge, the simultaneous use of these 3 biomarkers for the diagnosis and prognosis of CHD has never been investigated.

Thus, in this case–control study, we measured Cys-C, RBP, and IMA together with other laboratory parameters, and evaluated the individual and collective diagnostic and prognostic utility of Cys-C, RBP, and IMA for CHD. Thus, our findings can help with the early diagnosis, therapy, and prognosis prediction of patients with CHD.

## 2. Materials and methods

### 2.1. Participants

A total of 328 participants were enrolled in this case–control study, including 201 patients with CHD (case group) and 127 healthy individuals (control group). The case group included 127 males and 75 females with a mean age of 62 years (range: 55–70 years). All patients underwent CAG, which was performed by experienced cardiac interventional physicians. Angiography confirmed that 201 patients had significant CHD. The case group was further divided into the following subgroups^[15]^:

1. Acute myocardial infarction (AMI) group (n = 27): Patients admitted within 12 hours of symptom onset to the coronary intensive care unit of our hospital with AMI. AMI was confirmed based on typical symptoms and signs consistent with myocardial ischemia, such as chest pain or anginal equivalent that lasted more than 30 min; emerging ischemic ST–T changes, such as ST-elevation, ST-segment depression, or significant T-wave inversion in at least 2 contiguous electrocardiography leads; and increased levels of cardiac-related biomarkers of necrosis.
2. Stable angina pectoris (SAP) group (n = 22): Patients with SAP who were diagnosed with chest pain or similar angina at the time of exercise, had signs of myocardial ischemia after functional tests (myocardial perfusion imaging, stress echocardiography, and exercise electrocardiogram), and previously confirmed CAD, defined as prior myocardial infarction, coronary bypass graft, or percutaneous coronary intervention.
3. Unstable angina pectoris (UAP) group (n = 152): Patients with UAP, such as those with recent cardiogenic symptoms or resting angina, and normal cardiac troponin levels.

The control group consisted of 55 males and 72 females with a mean age of 52 (range: 45–62) years. These were healthy individuals who underwent physical examination at the Henan Provincial People’s Hospital and had no history of cardiovascular disease, hypertension, or diabetes. Liver and kidney function, myocardial enzymes, blood lipids, blood glucose, and other biochemical indicators in these individuals were normal.

This study was approved by the Ethics Committee of Henan Provincial People’s Hospital. All participants enrolled in this study were from the Henan Provincial People’s Hospital and provided written informed consent to participate.

### 2.2. CAG assessments

Coronary atherosclerotic burden was assessed using the Gensini score,^[16,17]^ which rated coronary lumen stenosis as 1 (1%–25% narrowing), 2 (26%–50% narrowing), 4 (51%–75% narrowing), 8 (76%–90% narrowing), 16 (91%–99% narrowing), or 32 (complete occlusion). Furthermore, the score was multiplied by an index based on the relevance of the lesion site in the coronary tree. For example, the left main branch was 5, proximal part of the left anterior descending branch (LAD) or left circumflex branch (LCX) was 2.5, middle part of the LAD was 1.5, and distal portion of the LAD or central part of the LCX was 1. The Gensini score is the total score for all coronary arteries. The patients were dichotomized into 2 groups based on whether their scores were >30 or ≤30 points. In addition, patients were separated into 2 groups according to their outcome: those with major adverse cardiovascular events (MACE) were classified as having poor outcomes, whereas those without MACE were considered to have good outcomes.^[18]^

Venous blood samples were collected from the control group in the morning of the postfasting period for medical examination. Venous blood samples from patients with CHD were collected from the antecubital vein after overnight fasting. A 6-mL plasma sample was drawn using ethylenediaminetetraacetic acid as an anticoagulant. Plasma samples were centrifuged for 15 min at 3000 × g, aliquoted within 30 min, and stored at –80 °C until use. All the samples were thawed once.

Cys-C and RBP concentrations were measured using commercial enzyme-linked immunosorbent assays (ELISA) (Ningbo Kongmei Biotechnology Co. Ltd., Ningbo, China) according to the manufacturer instructions. Absorbance was measured using a multimode detector (DTX880; Beckman Coulter, Fullerton, CA, USA). A microplate reader was used to measure the absorbance at 450 nm, with the wavelength correction set at 540 or 570 nm (DTX 880, Beckman Coulter). IMA was detected using a commercial kit (Ningbo Kongmei Biotechnology Co., Ltd.) and was measured by the reaction of residual free Co2 + with organic chromogenic substances after combining albumin with Co2 + to generate reddish-brown products. The IMA concentration was calculated after colorimetric comparison with standard substances at specific wavelengths. A Siemens ADVIA2400 automatic biochemical analyzer (Erlangen, Germany) was used to determine the levels of alanine aminotransferase, aspartate aminotransferase, blood urea nitrogen (BUN), creatinine (Cr), blood glucose, high-sensitivity C-reactive protein, low-density lipoprotein cholesterol (LDL-C), apolipoprotein (Apo) A1, ApoB100, total cholesterol (Chol), triglycerides (TG), and high-density lipoprotein cholesterol (HDL-C). SYSMEX XN-20 (Kobe, Japan) was used to detect hemoglobin (Hb) and white blood cells (WBC) in whole blood samples. All parameters were measured according to the manufacturer instructions.

### 2.3. Statistical analyses

Statistical analyses were performed using the SPSS for Windows 25.0 (IBM Corp., Armonk, NY, USA). The results of the measurement data are expressed as the median (interquartile range), and differences in the levels of indicators between groups were analyzed using the Mann–Whitney *U* test. Unconditioned multivariate logistic binary regression analysis was performed with CHD as the dependent variable, and Cys-C, RBP, IMA, and other laboratory parameters as independent variables. The diagnostic performance of each indicator detected individually or collectively was analyzed using receiver operating characteristic (ROC) curves, and the area under the curve (AUC), 95% confidence interval (CI), and standard error (SE) were calculated using SPSS 25.0. Survival curves (70-day) were obtained using the Kaplan–Meier method and compared using the log-rank test, performed using Prism 8.0 (GraphPad Inc., Boston, MA, USA). Prism software (version 8.0) was used to plot the curves. Two-tailed *P* values < .05 indicated that a difference was statistically significant.

## 3. Results

### 3.1. Baseline characteristic and univariable analysis for CHD

Of the 201patients, 126 were male (62.7%), and the mean age was 62.4 ± 11.04 years. A total of 152 (75.6%), 22 (10.9%), and 27 (13.4%) patients had UAP, SAP, and IMA, respectively. CAG revealed that 50, 43, 49, and 59 CHD patients had 1, 2, 3, and 4 cardiovascular blockages, respectively (see Table S1, Supplemental Digital Content, http://links.lww.com/MD/N415 that shows the results of univariable analysis for CHD). There were no significant differences in WBC, Hb, blood glucose, or TG levels between the case and control groups (*P > *.05). However, there were significant differences in age, sex, and BUN, Cr, HDL-C, LDL-C, and Chol levels between patients with CHD and the control group (*P* < .05) (Table S1, Supplementary Digital Content, http://links.lww.com/MD/N415).

**Figure 1.**
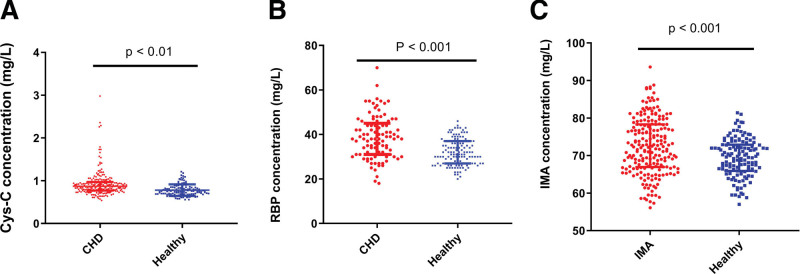
Comparison of Cys-C, RBP, and IMA levels between the case and control groups.

**Figure 2.**
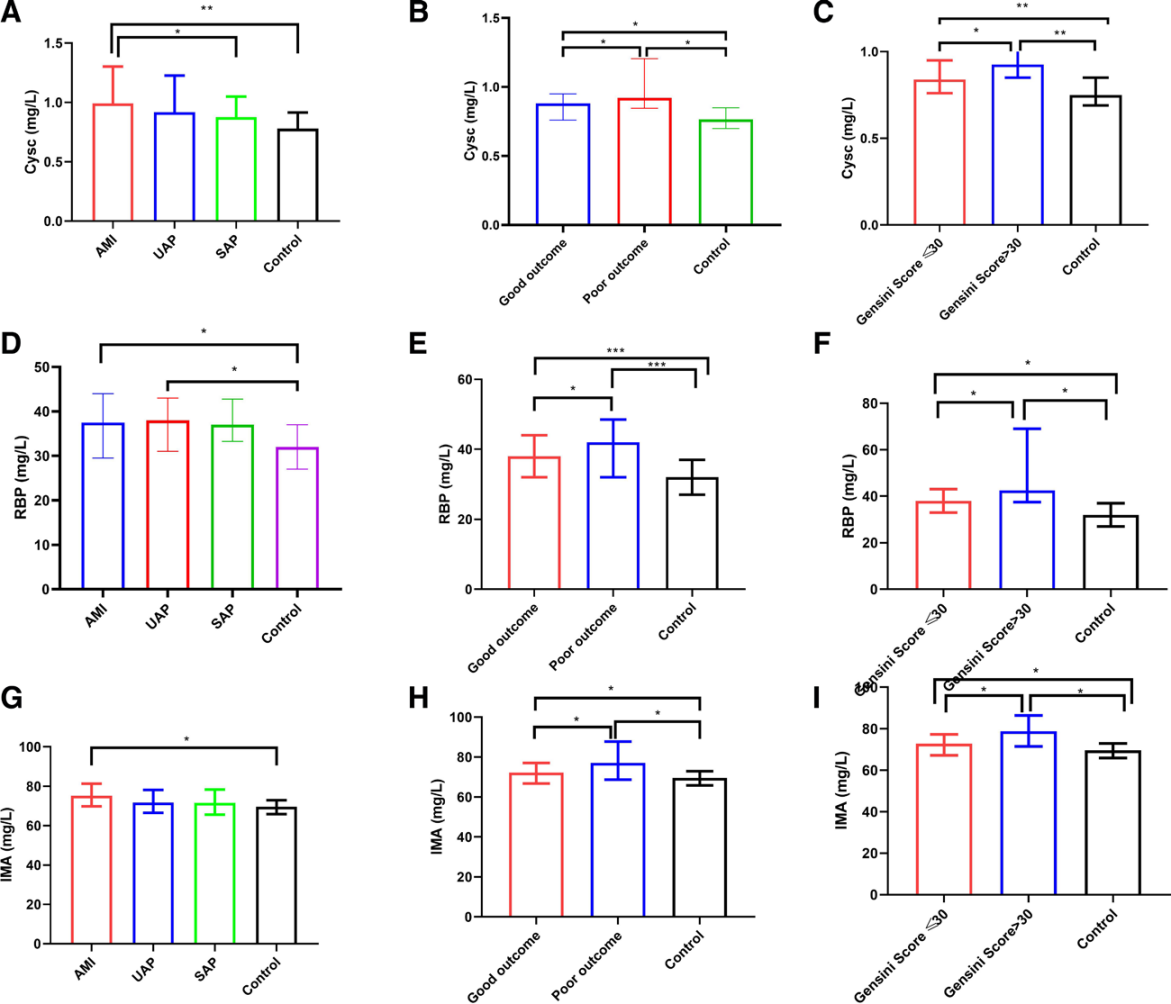
Comparison of Cys-C, RBP, and IMA levels among different groups.

**Figure 3.**
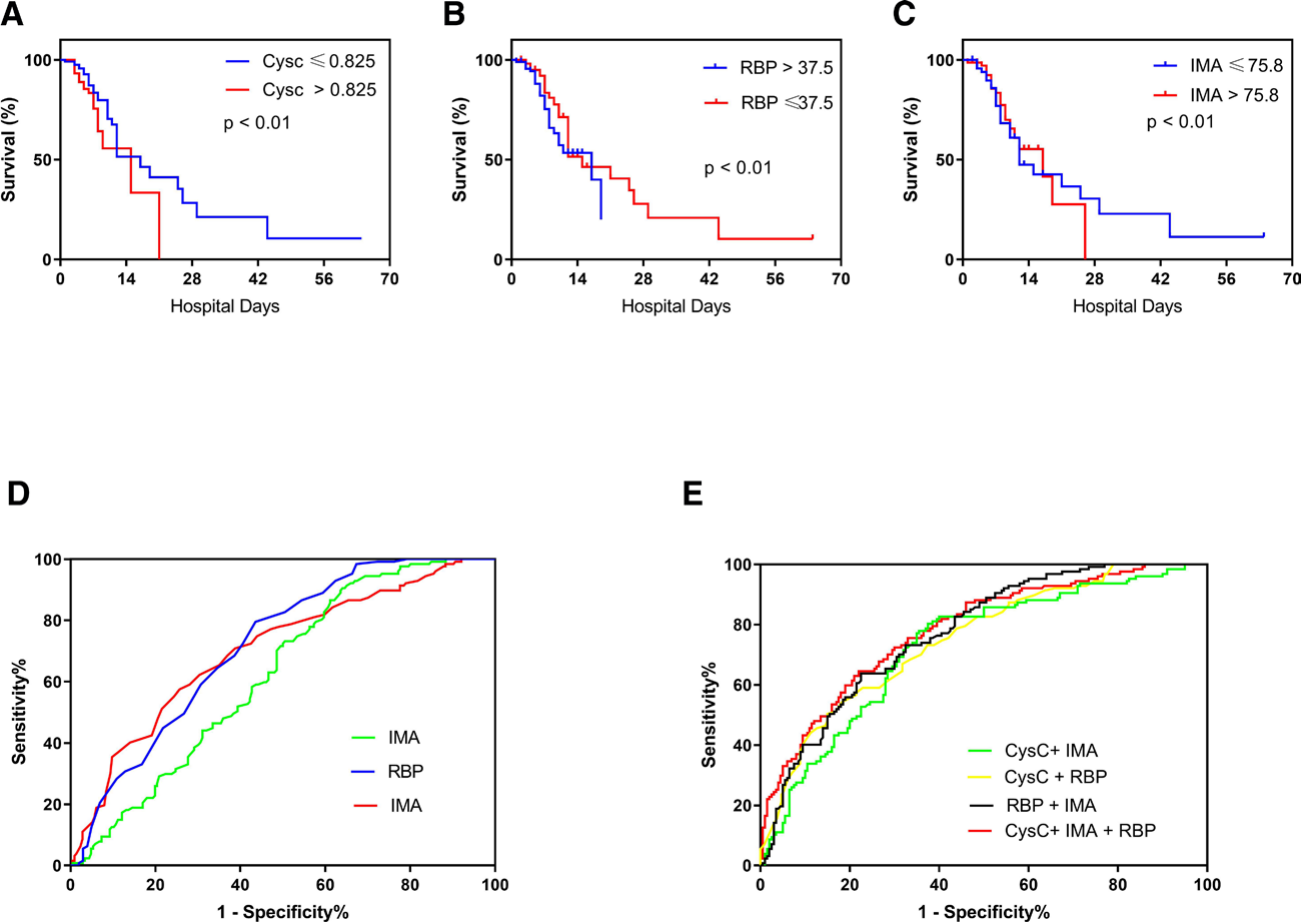
Survival and diagnostics curves with combinations of the 3 biomarkers: Cys-C, RBP, and IMA.

Cys-C, RBP, and IMA concentrations increased significantly in patients diagnosed with CHD compared with those in the control group (*P* < .05) (Fig. 1A–C). In addition, the Cys-C, RBP, and IMA levels were higher in patients with CHD and Gensini score > 30 with poor outcomes than in those with good outcomes and Gensini score ≤ 30 (see Table S1, Supplemental Digital Content, http://links.lww.com/MD/N415 that shows the results of univariate analysis for CHD, and Fig. 2A–I). Furthermore, the 70-day in-hospital survival analysis showed that Cys-C, RBP, and IMA levels could predict the survival outcomes of inpatients (Fig. 3A–C).

Multivariate unconditional logistic regression analysis was performed using CHD as the dependent variable; age and sex as the covariates; and Cys-C, RBP, IMA, and other laboratory parameters as the independent variables. The regression coefficient test showed statistically significant differences between the 2 groups for all 3 indicators (Cys-C, *P* = .002; RBP, *P* < .001; and IMA, *P* = .034). Therefore, Cys-C, RBP, and IMA were independent risk factors for CHD (see Table 2, Supplemental Digital Content, http://links.lww.com/MD/N415 that shows the results of the multivariate analysis of factors associated with CHD).

The diagnostic efficacy of each CHD indicator, tested separately and collectively Cys-C, RBP, and IMA are clinically significant predictors of CHD. Individual areas under the ROC curves (AUCs) for Cys-C, RBP, and IMA were 0.702, 0.707, and 0.625, respectively, indicating that the AUC for RBP was better. The combined AUC for Cys-C + RBP, Cys-C + IMA, and RBP + IMA were 0.745, 0.730, and 0.773, respectively. The AUC value of CHD detection using a combination of the 3 indicators was 0.783, indicating that the combined detection had a higher comprehensive diagnostic value than CHD detection using any of the 3 indicators individually. Moreover, triple combination therapy had a significantly higher diagnostic value (*P* < .001). Among the individual indicators, Cys-C had the highest sensitivity (70%) and IMA had the highest specificity (90.6%). Furthermore, for pairwise indicators, Cys-C + RBP had the highest sensitivity (78%) and Cys-C + IMA had the highest specificity (78%). When using the triple combination, the sensitivity and specificity were 78% and 74.6%, respectively (Tables 1 and 2, Fig. 3D and E, respectively).

**Table 1:** Results of receiver operating characteristic curve analysis.

Index	Cys-C	RBP	IMA	Cys-C + RBP	Cys-C + IMA	RBP + IMA	Cys-C + RBP + IMA
AUC	0.702	0.707	0.625	0.745	0.730	0.773	0.783
SE	0.030	0.028	0.030	0.027	0.028	0.025	0.026
95% CI	0.6 to 0.76	0.65 to 0.76	0.57 to 0.68	0.69 to 0.80	0.67 to 0.79	0.72 to 0.82	0.73 to 0.83
*P*	<.001	<.001	<.001	<.001	<.001	<.001	<.001

AUC = area under the curve, CI = confidence interval, Cys-C = cystatin-C, IMA = ischemia-modified albumin, RBP = retinol-binding protein, SE = standard error.

**Table 2:** The diagnostic efficacy of each CHD indicator, tested separately and collectively (%).

Index	Cutoff value	Sensitivity	Specificity	Positive predictive value	Negative predictive value
Cys-C	0.825	70.0	70.9	64.2	73.8
RBP	37.5	59.7	79.5	72.3	68.7
IMA	75.8	66.2	90.6	75.6	66.1
Cys-C + RBP	0.825 + 37.5	78	68.3	61	75.8
Cys-C + IMA	0.825 + 75.8	64.5	78	61.7	60.5
RBP + IMA	37.5 + 75.8	67.5	73.2	63.3	66.2
Cys-C + RBP + IMA	0.825 + 37.5 +75.8	78	74.6	73	71.8

Cys-C = cystatin-C, IMA = ischemia-modified albumin, RBP = retinol-binding protein.

## 4. Discussion

Although CAG is the standard protocol for the diagnosis of CHD, a common cardiovascular disease,^[5]^ its disadvantages (high cost, invasiveness, and allergy to contrast agents) impede its use in clinical practice. Peripheral blood biochemical investigations can provide simpler and more rapid indices for predicting and diagnosing CHD.^[18]^ While many studies have suggested the potential value of circulating Cys-C, RBP, and IMA as biomarkers for other diseases^[19–21]^ no previous study has investigated the prognostic role of these biomarkers in human CHD. We found that the levels of Cys-C, RBP, and IMA were all higher in patients with CHD than in the control group (*P < *.05), and all 3 were independent risk factors for CHD according to multivariate regression analysis, with RBP being the most significant individual indicator. However, the combination of these 3 indicators provided the highest AUC for CHD detection compared to any single or pairwise combination of the indicators. Moreover, serum Cys-C, RBP, and IMA levels may reflect the severity of CHD, although the serum Cys-C level was a better indicator than the other indicators. Such outcomes have not been previously reported.^[21]^

Cys-C has been widely used as an early indicator of renal dysfunction over the past few decades. Detailed studies have confirmed that Cys-C levels correlated positively with CHD and the degree of coronary artery stenosis.^[9]^ Serum Cys-C level was demonstrated as the main risk factor for CHD patients.^[10]^ This study showed that Cys-C levels in CHD patients were much higher than that in healthy participants, while logistic regression analysis revealed that the Cys-C level was an independent risk factor for CHD. As an individual indicator of CHD, the detection sensitivity of Cys-C was 70%, and the negative predictive value was 73.8%.

RBP, which is mainly synthesized by the liver, is involved in various metabolic pathways in vivo and plays important roles in vascular endothelial injury, atherosclerosis, and CHD. In the present study, significantly higher RBP levels were found in the CHD group than in the healthy control group. This finding was consistent with those of other studies.^[22]^ Logistic regression analysis showed that RBP is an independent risk factor for CHD. The AUC value of RBP for CHD diagnosis was 0.707, which was the highest among the 3 independent indicators in this study.

The IMA is a blood marker that is rapidly elevated and can be detected in vivo during ischemia and hypoxia. It appears earlier than other typical myocardial enzyme indices and thus holds considerable significance for the early diagnosis of acute CHD. Demirtas et al^[23]^ found that IMA can effectively detect UAP in patients with negative hypertension. In 2003, the American Food and FDA approved IMA as a clinical marker of early myocardial ischemia. The IMA level can be detected as a predictive indicator before coronary artery bypass surgery.^[24]^ Moreover, IMA is a more reliable predictor of postcardiac surgery risk than hyper-sensitive troponin.^[25]^ The results of this study showed that serum IMA levels increased significantly in patients with CHD as compared to the healthy control group. Logistic regression analysis revealed that IMA level was also an independent risk factor for CHD. As an independent indicator, the specificity of IMA for CHD detection was 90.6%, which was the highest among the 3 indicators considered in this study.

Furthermore, in this study, we used ROC curves to analyze the sensitivity and specificity of serum Cys-C, RBP, and IMA for the diagnosis of CHD. The AUC values of the pairwise combinations for CHD detection were higher than those of the single indicators. Among the pairwise combinations, the AUC value was the highest for RBP + IMA. Nevertheless, the AUC value when using the combination of all 3 indicators was higher than that for any other pairwise combination, indicating that the simultaneous detection of the 3 indicators had the greatest comprehensive value for the diagnosis of CHD. The sensitivity of the combined detection of the 3 indicators was 78%, which is consistent with the sensitivity of the Cys-C + RBP combination. Furthermore, the negative predictive value of the Cys-C + RBP combination was 75.8%, which was higher than that of the combined detection of the 3 indicators (71.8%), indicating that the combined detection of Cys-C + RBP may have good clinical applicability. We believe that these results can help implement early treatment, prevent or delay the progression of CHD, and effectively improve patient outcomes.

This study has some limitations. First, as this was a case–control study, matching bias could have occurred. Second, the sample size was small. In the future, we aim to collect more samples from inpatients with CHD and the control group participants to further analyze the relationship between CHD and Cys-C, RBP, and IMA, aiming for early diagnosis and therapy in these patients.

In summary, Cys-C, RBP, and IMA were independent risk factors for CHD. The simultaneous detection of Cys-C, RBP, and IMA may be helpful for differentiating clinical classifications, early diagnosis, and predicting CHD severity. However, these data need to be substantiated with a larger number of samples to ascertain the relationship between these 3 biomarkers and CHD comprehensively and conclusively.

## Acknowledgments

The authors gratefully acknowledge clinicians and patients for their participation in this study.

## Author contributions

**Conceptualization:** Youhua Yuan, Wenqian Tian, Xiaohuan Zhang.

**Data curation:** Youhua Yuan, Wenqian Tian.

**Investigation:** Xiaoxia Wei, Ya Zhu, Fengzhen Liu, Xiaohuan Zhang.

**Methodology:** Youhua Yuan, Wenqian Tian, Xiaohuan Zhang.

**Software:** Youhua Yuan, Wenqian Tian, Xiaohuan Zhang.

**Supervision:** Xiaoxia Wei, Ya Zhu, Fengzhen Liu, Xiaohuan Zhang.

**Validation:** Xiaoxia Wei, Ya Zhu, Fengzhen Liu.

**Visualization:** Xiaoxia Wei, Ya Zhu, Fengzhen Liu, Xiaohuan Zhang.

**Writing – original draft:** Youhua Yuan, Wenqian Tian.

**Writing – review & editing:** Youhua Yuan, Xiaohuan Zhang.

All authors approved the final version of the manuscript.

## Supplementary Material


