# Supplementary material for: Diagnostic and prognostic value of serum Cys-C, retinol-binding protein, and ischemia-modified albumin in patients with coronary heart disease: A diagnostic accuracy study
Source: Medicine (Baltimore). 2024 Aug 30;103(35):e39415. doi: 10.1097/MD.0000000000039415 (PMC11365651; doi:10.1097/MD.0000000000039415)
Supplement: Supplementary file 1 [file medi-103-e39415-s001.doc]

**Supplementary materials**

**Diagnostic and prognostic value of serum Cys-C, retinol-binding protein (RBP), and ischemia-modified albumin (IMA) in patients with coronary heart disease**

**Supplementary Table 1.** Results of univariable analysis for coronary heart disease (CHD)

| **Variables** | **CHD cases**  **(n = 201)** | **Controls**  **(n = 127)** | ***P*-value** |
| --- | --- | --- | --- |
| **Baseline information** |  |  |  |
| Age (years) (X ± S) | 62.4 ± 11.04 | 54.1 ± 12.99 | <0.05 |
| Sex (Men/Women) | 126/75 | 55/72 | <0.001 |
| WBC (×10^9^/L), (M, P25, P75) | 6.1 (5.1, 7.0) | 5.9 (4.9, 6.9) | >0.05 |
| Hb (g/L), (M, P25, P75) | 136.4 (127, 145) | 134 (126, 146) | >0.05 |
| Blood glucose (mmol/L) (M, P25, P75) | 4.8 (4.2, 5.8) | 4.7 (4.3, 5.0) | >0.05 |
| ALT (U/L) (M, P25, P75) | 23 (16, 29) | - | - |
| AST (U/L) (M, P25, P75) | 21 (18, 26) | - | - |
| SAP (n, %) | 22 (10.9) | - | - |
| UAP (n, %) | 152 (75.6) | - |  |
| AMI (n, %) | 27 (13.4) | - |  |
| **Laboratory parameters** |  |  |  |
| BUN (mmol/L) (M, P25, P75) | 5.2 (4.3, 6.2) | 4.6 (3.8, 5.4) | < 0.01 |
| Cr (μmol/L) (M, P25, P75) | 66 (55, 75) | 57 (57, 67) | < 0.01 |
| TG (mmol/L) (M, P25, P75) | 1.4 (1.0, 1.8) | 1.4 (1.0, 1.9) | > 0.005 |
| HDL-C (mmol/L) | 1.0 (0.9, 1.2) | 1.3 (1.1, 1.5) | < 0.001 |
| LDL-C (mmol/L) | 2.1 (1.5, 2.7) | 2.5 (2.1, 3.0) | < 0.001 |
| Hs-CRP (mg/L) | 1.1 (0.5, 5.6) | - | - |
| Chol (mmol/L) | 3.9 (3.2, 4.8) | 4.8 (4.2, 5.4) | < 0.001 |
| ApoA1 (mmol/L) | 0.9 (1.0, 1.2) | - | - |
| ApoB100 (mmol/L) | 0.8 (0.6, 0.9) | - | - |
| Cys-c (mg/L) | 0.86 (0.77–0.95) | 0.75 (0.69, 0.85) | < 0.001 |
| RBP (mg/L) | 37.9 (31.8–43.7) | 32 (27, 37) | < 0.001 |
| IMA (mg/L) | 72.2 (66.9–78.1) | 69.6 (65.9, 72.9) | < 0.001 |
| **Coronary angiography** | | | |
| **1 Blocked** | 50 | 0 | < 0.001 |
| **2 Blocked** | 43 | 0 | < 0.001 |
| **3 Blocked** | 49 | 0 | < 0.001 |
| **> 4 Blocked** | 59 | 0 | < 0.001 |

WBC, white blood cell count; Hb, hemoglobin; ALT, alanine aminotransferase; AST, aspartate aminotransferase; SAP, serum alkaline phosphatase; UAP, unstable angina pectoris; AMI, acute myocardial infarction; BUN, blood urea nitrogen; Cr, creatinine; TG, triglycerides; HDL-C, high-density lipoprotein cholesterol; LDL-C, low-density lipoprotein cholesterol; hs-CRP, high-sensitivity C-reactive protein; Chol, cholesterol; Apo, apolipoprotein; Cys-C, cystatin-C; RBP, retinol-binding protein; IMA, ischemia-modified albumin.

**Supplementary materials**

**Diagnostic and prognostic value of serum Cys-C, retinol-binding protein (RBP), and ischemia-modified albumin (IMA) in patients with coronary heart disease**

**Supplementary Table 2**. Results of multivariate analysis for factors associated with coronary heart disease

| **Risk factor** | ***P*-value** | **OR (95% CI)** |
| --- | --- | --- |
| RBP | <0.001 | 1.2 (1.1–1.4) |
| IMA | <0.001 | 1.1 (1.1–1.3) |
| Cys C | <0.02 | 100 (2.2–120) |
| Chol | <0.001 | 22.2 (4.3–115) |
| LDL | 0.007 | 21.2 (2.5–167) |

RBP, retinol-binding protein; IMA, ischemia-modified albumin; Cys-C, cystatin C; Chol, cholesterol; LDL, low-density lipoprotein; OR, odds ratio; CI, confidence interval
